# Work, life, and the gender effect: Perspectives of ACVIM Diplomates in 2017. Part 2—The intersection of personal life and professional career

**DOI:** 10.1111/jvim.15873

**Published:** 2020-08-19

**Authors:** Samantha L. Morello, Sara A. Colopy, Ruthanne Chun, Kevin A. Buhr

**Affiliations:** ^1^ Surgical Sciences University of Wisconsin‐Madison School of Veterinary Medicine Madison Wisconsin USA; ^2^ Department of Medical Sciences University of Wisconsin‐Madison, School of Veterinary Medicine Madison Wisconsin USA; ^3^ Department of Biomedical Informatics University of Wisconsin‐Madison, School of Medicine and Public Health Madison Wisconsin USA

**Keywords:** family, gender, professional, work‐life balance

## Abstract

**Background:**

In the field of veterinary surgery, women neither marry nor have children at the same rate as men, and those who do may experience more career disruption as a result. The American College of Veterinary Internal Medicine (ACVIM) is 1 of the few areas of specialized medicine that is predominantly female; it is unknown if such a demographic shift would produce a different environment for individuals cultivating their personal life.

**Hypothesis/Objectives:**

To report data regarding subjective and objective aspects of the intersection of the personal and professional lives of Diplomates of the ACVIM.

**Sample:**

Eight hundred ninety‐six surveys (781 completed) of ACVIM Diplomates, including cardiology, large and small animal internal medicine, neurology, and oncology.

**Methods:**

An 82‐item online survey was distributed to ACVIM Diplomates via Diplomate college listservs. Participation was voluntary.

**Results:**

Men were more likely to be married and have children than were women. Women had or adopted their first child at a later career stage compared with men, and agreed more strongly that career stage was an influential factor in family planning. Those with children worked fewer hours compared with those without, and this effect was greater among women. Women were more likely to require external childcare, but most men and women shared childcare responsibilities equally outside of working hours.

**Conclusions and Clinical Importance:**

The intersection of personal and professional life differs between men and women in the ACVIM, which may create different needs, preferences, or barriers to work‐life balance in the workforce.

**Abbreviations**
ACVIM: American College of Veterinary Internal Medicine
ACVS: American College of Veterinary Surgeons
LAIM: large animal internal medicine
SAIM: small animal internal medicine

## 1 INTRODUCTION

Veterinary medicine has evolved over the past 40 years as rapidly as any other high‐level profession of note. This evolution includes the use of modern science and technology, increasing specialization within fields, as well as improving management and organization of hospitals and practices. It has become increasingly common for specialty practitioners to gravitate toward private practice rather than academia—a choice based on a variety of factors, including professional, economic, and personal reasons. In a time when more and more choices are available, Diplomates may have more flexibility in prioritizing the needs of their personal lives over their professional ones.

Both veterinary and human medical programs currently report a female majority in their graduate training programs.^1^, ^2^ However, veterinary medicine has changed more dramatically than human medicine. In 2019, 50.5% of matriculants to United States medical schools were women, compared with 80.5% of those enrolling at US veterinary medical colleges.^1^, ^2^ In this way, veterinary medicine can serve as a model for other high‐level professional groups, providing an example of what happens when a field requiring multiple years of graduate and postgraduate training shifts to a predominantly female demographic. The American College of Veterinary Internal Medicine (ACVIM) could serve as a particularly good model for considering the features of a feminized medical profession because it is a large field, comprised of many specialties, each of which reports a female majority membership.^3^

The professional lives of male and female veterinarians differ substantially, and practice setting is a major component of this difference.^3^, ^4^ Furthermore, the personal and professional lives of veterinary surgeons intersect, and those experiences may differ between men and women.^5^ In contrast to the ACVIM, the American College of Veterinary Surgeons (ACVS) is a predominantly male specialty college. The culture of practice of specialties within the ACVIM may foster an environment more attractive to women in the profession. Conversely, the culture of practice may have adapted to a feminized workforce, and that similar assessment of the intersection of professional and personal lives of ACVIM Diplomates may differ. Although differences between personality traits and professional interests play a considerable but unmeasurable role in determining career path, contrasting the experience of ACVIM Diplomates with that of ACVS Diplomates will broaden the knowledge base for what life as a veterinary specialist entails.

Our objectives were to collect and consider data regarding the reciprocal effects of a career and a personal life for ACVIM Diplomates. Topics including personal relationships, family planning and balance, and the subjective experience of how these elements integrate were explored using a survey questionnaire. Our aims include providing relevant, objective, and statistically appropriate information to those in the field to inform decision making and guide practice management.

## 2 MATERIALS AND METHODS

### 2.1 Study population

The study group comprised ACVIM Diplomates in good standing as of February 2017 who were subscribed to ≥1 listservs serving the specialties of large animal internal medicine (LAIM), small animal internal medicine (SAIM), cardiology, oncology, and neurology. The size and gender demographic for each college specialty are reported elsewhere,^3^ but the number of Diplomates subscribed to each listserv was unknown. The institutional review board at the University of Wisconsin‐Madison waived formal review of the protocol because of the nature of the study in accordance with Federal Regulation 45 CFR 46.102(d).

### 2.2 The survey

An 82‐item questionnaire (Supplementary Item 1) collected objective and subjective data about the professional and personal lives of Diplomates, and was administered using an online platform (Qualtrics©, Provo, Utah). Broad details regarding the surveyed areas are reported elsewhere.^3^ This manuscript addresses responses to questions directed toward the respondents' personal life and family (marital status, children, childcare arrangements, age at first child), the interdependent effects of career and family, and any perceived effect of gender on these topics. All data collected was anonymous and automatically entered into a computerized database for analysis. Responses were collected over the course of 1 month, with 3 email prompts for participation.

### 2.3 Study analysis

Response counts and percentages (for non‐missing responses) were calculated. For continuous variables with categorical coding (eg, age, hours worked per week), category midpoints were used and treated as continuous values for analysis. For group comparisons of categorical responses, *P*‐values were calculated using chi‐squared tests (or using Cochran‐Mantel‐Haenszel tests for adjustment by a single categorical covariate or logistic regression for adjustment by single continuous covariates or multiple covariates). For group comparisons of continuous responses (including category midpoints for categorically coded continuous variables), linear models were used. Smoothed estimates of percentages of respondents having children as a function of Diplomate year were fit using a generalized linear (logistic) model using a B‐spline basis for Diplomate year and a main effect for gender; separate models were fit for ≥1 and ≥2 children. For personal income, which was subject to right censoring at $400 K, regression models with adjustments for covariates were fit using parametric survival analysis under a Weibull distribution assumption. Although such analysis is most commonly used for survival data, it is also appropriate for other types of data subject to right censoring. All statistical analysis was performed using R version 3.6.3 (R Core Team, R Foundation of Statistical Computing, Vienna, Austria, 2020)

## 3 RESULTS

### 3.1 Personal demographics

A total of 896 individuals responded with 781 (87.2%) completing the survey. All results described below are based on analyses of completed surveys. A sensitivity analysis of data was conducted that included incomplete surveys, and results were similar to all results presented here. Across all survey responses analyzed, the percentage of missing responses was ≤5% Response by specialty was: SAIM, 294 (37.8); LAIM, 175 (22.5%; equine, farm animal, or mixed); cardiology, 84 (10.8%); neurology, 95 (12.2%); and oncology, 130 (16.7%) with 3 respondents not providing a specialty. Of those providing information regarding gender (n = 776), 25.3% (n = 196) identified as male, 74.6% (n = 579) as female, and 0.1% (n = 1) as other.^3^

### 3.2 Marital status and spousal information

Overall, 76.9% (n = 598) of Diplomates indicated they were married or in a domestic partnership. At the time of survey, 5.3% (n = 41) were separated or divorced, 0.1% (n = 1) were widowed, and 17.7% (n = 138) were single (and never married). Significant differences were found in relationship status between men and women after adjusting for age (*P* = .002); men were more likely to be married or in a domestic partnership (n = 169, 86.2% versus n = 426, 73.6% of women), and women were more likely to be single and never previously married (n = 117, 20.2% versus n = 21, 10.7% of men). Men were more likely to be with a partner who was also a veterinarian compared with women (n = 86, 48.0% versus n = 130, 27.4%, *P* < .001). No differences were found in the proportions of those who were ever married or partnered versus single by specialty (*P* = .89) or practice type (academia versus private practice, *P* = .64).

### 3.3 Number and timing of children

**FIGURE 1:**
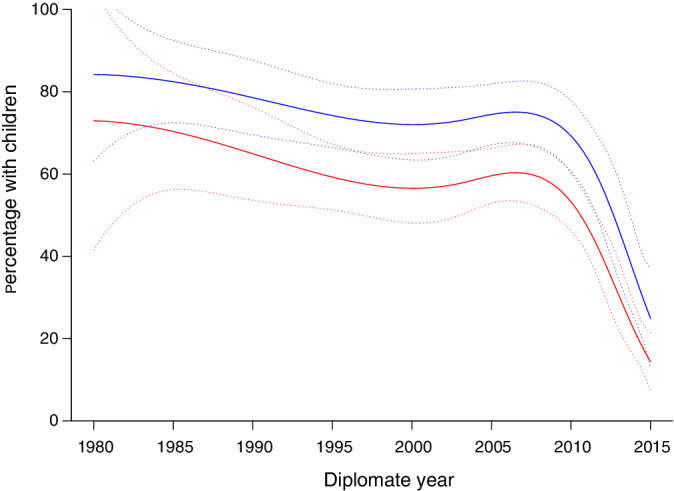
Smoothed estimates, by gender, of percentages of respondents with children as a function of Diplomate year. Curves for men (blue) based on n = 133 and n = 62 men with and without children respectively, and women (red) based on n = 265 and n = 304 women with and without children respectively are given. Estimated mean percentages (solid curve) and 95% pointwise confidence intervals (dashed curves) are given

Overall, 51.7% (n = 402) of respondents reported that they had at least 1 child; this proportion was significantly higher among men (n = 133, 67.9%) than women (n = 267, 46.2%; *P* < .001) and the difference remained significant after adjusting for age (*P* < .001, Figure 1). Even within the subset of respondents >35 years of age who identified themselves as “not single,” women were still less likely to report having children as compared with men (n = 234, 62.6% versus n = 126, 80.8%; *P* < .001). No difference was found in the proportion of those with children between those in academia versus private practice. Among respondents reporting having children, the mean number of children was 1.9.

**FIGURE 2:**
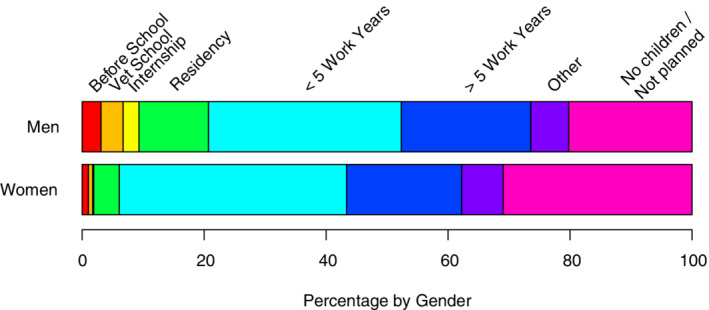
Strip plot representing responses by gender to the question “When during your career did you/do you plan to have or adopt your first child?” Bar segment lengths reflect percentage of responses out of those with a non‐missing response, and answers are categorized by color (n = 19, men; n = 572, women). Possible answer choices, from left to right, included before (red, far left) or during (orange) veterinary school, during internship (yellow), during residency (green), during (sea green) or after (dark blue) the first 5 years of full‐time work, other (purple), or not planning on having children (pink, far right)

Respondents reported having or adopting their first child at an average age of 34.3 years (n = 393), with no difference by gender. However, a significant difference was found between male and female Diplomates in the timing, relative to career stage, of having or adopting their first child. Women respondents had their first child later in their career than did men (*P* < .001; Figure 2). In particular, among those having children, 31.7% of men (n = 40) versus 12.0% of women (n = 31) had their first child during or before completing their residency program. More women (n = 212, 47.2%) than men (n = 47, 28.7%) strongly agreed that their stage of career (in training versus employment, or number of years practicing) played or would play an important role in family planning (*P* < .001).

**FIGURE 3:**
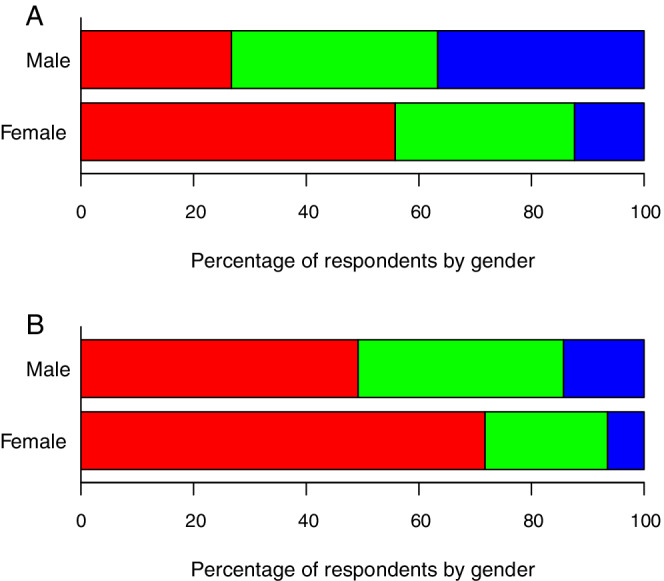
Responses by gender to the questions (A, top panel, for those with children: male, n = 131; female, n = 267) “Do you feel that having a family has had an impact on your career?” and (B, bottom panel, for those without children: male, n = 63; female, n = 308) “Do you feel that having a family will impact/would have impacted your career?” Possible answer choices, from left to right, included “negatively” (red, far left), “neutral” (green), or “positively” (blue, far right). Bar segment lengths reflect percentage of responses out of those with a non‐missing response

### 3.4 Interrelationship of children and professional demographics

The ACVIM Diplomates worked an average of 40 to 49 h/wk and hours worked varied by practice setting and, to a lesser extent, by specialty and by gender.^3^ A difference was observed in the number of hours worked between those with children and those without (median, 40‐49 versus 50‐59 h/wk; *P* < .001). In particular, 20.2% of respondents with children (n = 81) indicated that they worked <40 h/wk compared with 9.3% of those without children (n = 35), and this difference was higher for women than men (n = 62, 23.4% versus n = 29, 9.3% for women with and without children, respectively, and n = 19, 14.3% versus n = 6, 9.5% for men with and without children, respectively; *P* < .001). Diplomates who either had or were planning to have children were asked if having children had or would have an effect on the number of hours worked per week. Overall, 75.8% of respondents (n = 429) indicated that their working hours had decreased or would decrease to some extent, with a significant difference by gender (n = 338, 82.8% of women versus n = 91, 58.0% of men, *P* < .001). This difference was most notable among those who responded that their working hours would decrease to between 20 and 40 h/wk (n = 10, 6.4% of men versus n = 120, 29.4% of women).

Respondents with children were asked whether having children affected the type of practice or career path they had chosen. Some examples provided within the text of the question included academia versus private practice, associate versus practice owner, specialty practice versus general practice, or large animal versus small animal specialty. Responses differed significantly by gender with 36.4% of men (n = 48) and 48.7% of women (n = 130) indicating their career paths had been affected (*P* = .02), but answer choice options did not delineate specifically how this effect manifested itself. Responses did not vary by specialty type. Diplomates who either had or planned to have children were asked whether having children had or might influence a decision to pursue a career path outside of clinical practice. Almost half (48.3%) of females (n = 202) versus 31.7% of males (n = 51, *P* = .002) indicated that they agreed or strongly agreed with this statement.

We assessed the association between having children and measures of professional success. Although respondents with children were more likely to be more advanced in their careers, these differences disappeared after adjusting for age and relationship status. Similarly, although respondents with children reported higher incomes than those without, the difference was not significant after adjusting for key covariates, including gender age, Diplomate year, practice type, and employment status.

### 3.5 Work flexibility and responsibilities in the home

Given the competing priorities of work and home life, Diplomates were asked about flexibility in their work schedules. No differences in responses were found by gender either overall (*P* = .58) or among those with children (*P* = .37). In particular, no differences were found in the proportion of men versus women who felt his or her partner had more flexibility than he or she did themselves, either overall (n = 103, 56.3% versus n = 289, 58.6%), or among those with children, (n = 80, 60.6% versus n = 144, 55.8%). More men than women indicated that their partner stayed at home full time (n = 26, 14.2% versus n = 29, 5.9%) or had a job that was either part‐time or otherwise allowed for substantial time off (n = 28, 15.3% versus n = 45, 9.1%). More women than men indicated that their partner had more flexibility with his or her or their work hours (n = 179, 36.3% versus n = 36, 19.7%).

Significant differences were observed by gender in provision of childcare (*P* < .001). More female (n = 222, 83.1%) than male (n = 63, 51.2%) Diplomates indicated that they currently or previously required external childcare (outside of an immediate family member), and more men (n = 39, 31.7%) than women (n = 20, 7.5%) indicated that their partner or spouse stayed home with the children. Respondents with children also were asked about childcare outside of regular working hours. Significant differences existed between men and women (*P* < .001), with 30.3% of women (n = 80) versus 3.2% of men (n = 4) reporting themselves as the primary caregiver, whereas 3.0% of women (n = 8) and 21.4% of men (n = 27) reported that his or her spouse filled this role. The majority of both men (n = 74, 58.7%) and women (n = 147, 55.7%) reported sharing this responsibility equally with their partners.

Respondents with children were asked a series of questions regarding parental leave; for those with multiple children, the maximum total time taken for 1 child was used for analysis (n = 360). Women took significantly more child‐related leave (mean, 16.3 weeks; n = 257) compared with men (mean, 3.3 weeks; n = 101; *P* < .001), a difference that remained significant after adjusting for practice type and species. Men reported taking no weeks of parental leave 13.9% (n = 14) of the time, whereas women reported taking no leave 2.7% (n = 7) of the time. Overall, 28.0% (n = 203) of respondents indicated that their parental leave was fully paid, whereas 43.9% (n = 313) reported this time as unpaid and 28.1% (n = 204) as partially paid; there was no evidence of differences in these proportions by gender. Those employed in an academic setting were far more likely to report that parental leave was fully compensated compared with those working in private practice (n = 141, 58% versus n = 50, 12%, *P* < .001). The average number of fully or partially paid weeks of parental leave was 4.9 weeks and 7.5 weeks, respectively. For those receiving compensation for parental leave, the most common coverage category was “compensation specifically designated for parental leave,” followed by “vacation time.” Overall, 34.8% (n = 135) of respondents felt that the time allowed for parental leave was adequate; this percentage did not vary by gender (*P* = .45), but agreement was somewhat higher for those in academia compared with those in private practice (n = 53, 42.1% versus n = 70, 31.2%, *P* = .03). The majority of individuals felt that their supervisors (n = 236, 66.3%) and colleagues (n = 245, 68.4%) were supportive of their time taken for parental leave.

### 3.6 Subjective measures of personal life and career balance

Diplomates were asked about the effects that their career may or may not have had on their personal relationships. Overall, 15.4% (n = 117) felt that their career had positively impacted their relationships, whereas 48.0% (n = 364) reported a negative effect. Women were somewhat less likely than men to report a positive effect (n = 77, 13.6% versus n = 39, 20.5%, *P* = .05).

Diplomates were asked to subjectively assess the impact of family on career. Among Diplomates with children, women were significantly more likely than men to perceive a negative impact of having a family on their professional career (n = 149, 55.8% versus n = 35, 26.7%) and were less likely to perceive a positive impact (n = 33, 12.4% versus n = 48, 36.6%; *P* < .001; Figure 3). Respondents without children were asked what sort of impact they expected having children would have on their career women were significantly more likely to expect a negative impact than were men (n = 221, 71.8% versus n = 31, 49.2%; *P* = .002; Figure 3). Overall, the beliefs of those without children were significantly more negative than the actual experiences of those with children (*P* < .001).

Diplomates with children were asked for their subjective assessment of the effect of taking time from work for parental leave on their ability to reach their career goals. Almost 14% (n = 14) of men and 2.7% (n = 7) of women responded that they did not take parental leave. Among the remaining respondents, 6.9% of men (n = 6) felt that parental leave had affected their career progression, compared with 18.9% of women (n = 46; *P* < .001).

Participants who indicated they had children were asked whether maintaining their career had negatively impacted their participation in family life, especially with respect to raising children. The majority of respondents agreed or strongly agreed (n = 276, 68.7%) that it had. This proportion was higher for women than for men (n = 201, 75.3% versus n = 74, 55.6%; *P* < .001).

## 4 DISCUSSION

The current professional demographics of the different specialty groups within the ACVIM, and some objective and subjective measures of professional success and climate among men and women, and in different age groups are described elsewhere.^3^ Here, we consider the same population to summarize elements of life outside of the work environment, and assess the conflicting effects of personal life and a demanding professional career. Although fewer differences appear to exist among the various specialties in this regard, it is clear that male and female Diplomates experience distinct differences within these spheres. Outlining those discrepancies and considering where barriers and benefits might exist will be useful to current and future ACVIM Diplomates, as well as to those seeking to hire and retain them.

Among ACVIM Diplomates, men were more likely to be married than were women, and women were more likely to report being single and not previously married. These findings were significant even after accounting for the age difference between those populations, and were consistent with observations among Diplomates of the ACVS. Men were nearly twice as likely as women to be married to another veterinarian; this finding suggests the possible presence of an imbalanced “marriage market” created by current demographics in veterinary schools, where women have been the majority for over 30 years.^1^ Although no difference was found in divorce between the genders, an accurate rate could not be calculated because it is assumed that some proportion of those who had been divorced were remarried, and responded to the survey in the latter category. Only 5.3% of Diplomates responded that they were separated or divorced, which is in contrast to a finding among US medical physicians, where overall divorce prevalence was 24.3% and the number divorced at the time of survey was 7.7%.^6^ This same study showed a higher prevalence of divorce associated with longer weekly work hours among female physicians, and also among other professions such as dentists, pharmacists, lawyers, and health‐care executives. The supposition is that the stress and demands of the medical profession contribute to high divorce rates.^7^ Although the dataset provided in our study cannot be compared to that presented previously, the relatively small number of those reporting divorce, and the lack of an effect of gender on divorce, provides an encouragingly positive review for the intersection of marriage and professional life for the ACVIM, and its predominantly female workforce.

Predictably, the majority of men and women reported that their career had a negative effect on their personal relationships, with women being less likely to report a positive impact than men. This finding is intuitive, because the time and space the job requires are finite commodities. However, over half of all Diplomates indicated that their partner had more flexibility in their work schedule (by virtue of scheduling, part‐time work, or working from home), and that proportion was higher among respondents with children. This finding suggests that ACVIM Diplomates may benefit from relationships in which their life partners have jobs that allow for a flexible work schedule, allowing for adaptation to the taxing schedules associated with clinical care provision. For some life partners, this situation might present an unequal burden of compromise. This imbalance could be especially challenging for women, who were less likely to have a stay‐at‐home spouse or were subject to the pressure of maintaining traditional gender roles.

Fewer women had children than did men, regardless of the constraints of age or relationship status. This finding did not vary between practice types or specialties. This gender discrepancy is consistent with data observed among ACVS Diplomates, including the average age at which women had children (approximately 34 years of age).^5^ The decision of whether or not to have children is complex and personal, and largely outside the scope of our analysis. However, the smaller number of women having children coincides with a lower marriage rate compared with men and an average age of 34.3 years for women having their first child. This finding is especially important, because it estimates that almost half of female ACVIM Diplomates will have their children after 35 years of age, which is considered advanced maternal age, and associated with decreased fertility and increased risk of birth defects and other pregnancy‐related complications.^8^

Other observations made regarding family planning include the finding that women had children later in their career compared with men. Specifically, almost 33% of men had their first child before they completed residency training, as compared with only 12% of women. Women also were more likely to indicate that career stage had influenced family planning; the competing requirements of medical training and beginning a family may be more challenging for women. Anecdotally, it has become more common for female veterinary students, interns, and residents to combine these endeavors and, although institutional policies on pregnancy and lactation are rare,^9^ increasing attention has been paid toward amending this issue. Instead, women with children more often took time out during the early stages (first 5 years and beyond) of their careers to have children and also reported substantially more parental leave time than did men (average of 16.3 versus 3.3 weeks). Furthermore, 44% of respondents indicated that their parental leave was unpaid, and this forfeit is proportionally higher when taken out of the income generated during the postresidency years versus during veterinary school and training programs.

Policies regarding parental leave, including those referable to the Family Medical Leave Act (FMLA) vary by state, by institution, and across practice environments, and so too do the compensation strategies applied to those periods.^9^, ^10^ It was discouraging, yet unsurprising, that a minority of individuals received full compensation for their parental leave, and a much larger proportion reported the time as unpaid. Notably, those employed in academia were more likely to report fully compensated parental leave (58% compared with only 12% reporting fully compensated leave in private practice). Large organizations, such as universities, are more likely to have well‐established policies in place for prolonged periods of absence, and often allow serial banking of hours used for sick leave and vacation, which can be used later for extended periods of parental leave. Smaller private practices may not have similarly established protocols, and federal law exempts organizations with <50 employees from FMLA regulations.^10^ Furthermore, our finding that there were fewer specialists per specialty section in private practice, compared with academia, demonstrates the limited flexibility practices might have in developing policies that prioritize long periods of compensated leave for staff. However, >40% of those in academia were not fully compensated for their leave, highlighting the limitations to these policies, and their effect on personal income across all practice environments. Women, on average, reported 16.3 weeks (652 hours) of maternity leave, in our survey. In academia, many utilize banked sick leave and vacation for part or all of their leave time taken; some individuals may not have sufficient hours to cover leave, some may exhaust their compensated leave on their first child leaving too few hours for a second child, others may choose not to deplete banked leave and select to take unpaid leave instead, whereas still others may experience a variation in institutional policy on banking hours. Also, respondents may have interpreted answer choices such as “fully paid” or “partially compensated” differently, because utilizing one's own vacation time is not the equivalent of receiving paid family leave. Currently, only 6 states (California, Connecticut, Massachusetts, New Jersey, New York, Oregon, and Rhode Island) and Washington, DC, require paid family leave through FMLA. Overall, our results highlight 2 main points: first, that academia may offer better opportunity for consistent compensation during prolonged parental leave, and second and more importantly, that marked variation still exists in availability or accessibility to compensation for parental leave, or even in the language used surrounding those approaches. This variability may create confusion for young parents, for potential hires, and even for those reading our findings. Ultimately, we identify a very clear intervention point for both employers and employees, because seeking to improve the creation and transparency of policies, or more critical consideration of the various options among settings during a job search, could mitigate some of this difficulty for all.

The first 5 years of a specialty career are considered a time of critical professional development, during which clinical skills are solidified, a client base is built, research programs are started, with the resulting confidence and stability translating into improved performance. Although the leave period is finite, the residual effects of a hiatus and the ongoing responsibilities associated with raising children are not. Among respondents, women indicated greater need for external childcare as compared with men, suggesting increased financial and emotional stress, and possibly requiring a reduction in working hours. Women also reported being the primary caregiver to their children outside of working hours more frequently than did men, although the majority of both men and women shared this responsibility equally. The economic effect of women having children during their early professional years is described as the “motherhood penalty,” wherein women have been shown to suffer a 4% decrease in income associated with each child.^11^ Although children, as a specific covariate, did not appear to be associated with an income discrepancy, Diplomates with children reported working fewer hours per week, and this reduction was more frequent among women than men (23% versus 14%). Even among those without children, women were more likely than men to respond that they would decrease their working hours after having children. In private practice, income is clearly linked to number of hours worked. Therefore, it is reasonable to consider the effects that childbirth and young children might have on those early career stages both in a quantitative (income) and qualitative (advancement) manner.

Although being a parent may be a cause for the gender‐related advancement gap, likely other variables (eg, hours worked, employment status, relationship status) are responsible for this difference as well.^11^, ^12^ The Pew Research Center survey of working parents identified that the majority (65%) of mothers experienced a major career interruption (ie, decreasing working hours, taking significant time off, quitting a job, or turning down a promotion) in order to care for their family.^13^ Fortunately, longitudinal studies examining the longer‐term consequences of the “motherhood penalty” on career success indicate that the effects of having children on measures of occupational status and prestige attenuate over a period of 20 to 30 years. Career success can be challenging to define and measuring effects on hypothetical goals is even more fraught. Hence, we considered the subjective interrelationship of career and family. Almost 1 in 5 female respondents, compared with 1 in 14 males, believed that parental leave negatively affected their career, and 56% of female Diplomates reported that their careers had been negatively impacted by having children. Although the majority of both men and women who did not have children at the time of the survey believed that children would have a negative effect on their careers, this overall sentiment was far more pessimistic than the opinion of those who already had children. This discordance between anticipation and reality indicates either systemic bias regarding childbearing, lack of transparent conversation and education about being a parent, or both. This finding is similar to survey findings of senior residents and early career faculty medical physicians in the field of surgery about barriers to advancement where residents were twice as likely to suspect that childbearing would be a career barrier.^14^ Still, half of women reported experiencing a struggle with this balance. Development of policies in professional programs, as well as increased opportunities for positive and supportive discussion and dialogue within the workplace, would help combat any negative perception that may be associated with having children and a successful career.

Although we identified differences between male and female Diplomates who had children and those who did not, our study had some important limitations to consider in how some results are interpreted. Alterations in work schedules and personal responsibilities are, at least in part, a choice. Differences identified are often the result of those individual choices, albeit at times they may be difficult ones. The conscious prioritization of work versus family was not investigated, and it is likely that most people prioritize family. Also, in our study, children were the only dependents investigated. A more accurate scope of family would have included aging parents or other relatives who cannot care for themselves. In failing to account for such individuals, our results may present some bias against those definitions of family. It was also noted in the first part of our study that 776 individuals reported their gender.^3^ This data was solicited at the end of the study in an attempt to minimize response bias by avoiding sensitizing participants to the effect of gender. However, due to the nature of many of our questions, doing so was likely an impossibility. The overall completion rate was 87% and questions could be left unanswered at will by participants at any time. Therefore, determining a gender‐based participation bias, or other participation bias, is difficult and since not all questions represented identical datasets of respondents, response numbers should be considered in addition to percentages when interpreting our findings. Additional limitations relevant to the nature of our study design, such as the existence of response bias, and to the sample population, such as the paucity of racial diversity, are addressed elsewhere, but are critical to consider in the overall interpretation of our results.^3^

The ways in which personal life and career intersect for Diplomates of the ACVIM overall were strikingly similar to observations for Diplomates of the ACVS.^4^, ^5^ Beyond the technical occupational differences between the 2 colleges, the requirements and climates of training programs and employment environments are likely similar, because Diplomates of both colleges often work together. Although the majority of ACVIM Diplomates are women, and the majority of ACVS Diplomates are men, we did not identify differences in their experienced work environment. We suggest that a paradigm shift in workplace management and culture, structured more specifically around the needs of a female workforce, is critical in order to address the barriers and gender gaps that have been reported across various disciplines of veterinary medicine.^3^, ^4^, ^5^, ^9^

## CONFLICT OF INTEREST DECLARATION

Authors declare no conflict of interest.

## OFF‐LABEL ANTIMICROBIAL DECLARATION

Authors declare no off‐label use of antimicrobials.

## INSTITUTIONAL ANIMAL CARE AND USE COMMITTEE (IACUC) OR OTHER APPROVAL DECLARATION

Authors declare no IACUC or other approval was needed.

## HUMAN ETHICS APPROVAL DECLARATION

Authors declare human ethics approval was not needed for this study.
